# Modulating lncRNA SNHG15/CDK6/miR-627 circuit by palbociclib, overcomes temozolomide resistance and reduces M2-polarization of glioma associated microglia in glioblastoma multiforme

**DOI:** 10.1186/s13046-019-1371-0

**Published:** 2019-08-28

**Authors:** Zhenzhe Li, Jixing Zhang, Hongshan Zheng, Chenlong Li, Jinsheng Xiong, Weiliang Wang, Hongbo Bao, Hua Jin, Peng Liang

**Affiliations:** Department of Neurosurgery, Harbin Medical University Cancer Hospital, No.150 Haping Road, Nangang District, Harbin, Heilongjiang 150001 People’s Republic of China

**Keywords:** Temozolomide resistance, Glioblastoma, Microglial cells, lncRNA SNHG15/miR-627-5p/CDK6 signaling, Palbociclib

## Abstract

**Background:**

Accumulating evidence demonstrates the oncogenic roles of lncRNA (long non-coding RNA) molecules in a wide variety of cancer types including glioma. Equally important, However, tumorigenic functions of lncRNA in glioma remain largely unclear. A recent study suggested lncRNA SNHG15 played a role for regulating angiogenesis in glioma but its role in the tumor microenvironment (TME) was not investigated.

**Methods:**

First, we showed that SNHG15 was upregulated in GBM cells and associated with a poor prognosis for the patients of GBM using public databases. Next, we collected temozolomide sensitive (TMZ-S) and resistant (TMZ-R) clinical samples and demonstrated that co-culturing TMZ-R cells with HMC3 (microglial) cells promoted M2-polarization of HMC3 and the secretion of pro-GBM cytokines TGF-β and IL-6.

**Results:**

Comparative qPCR analysis of TMZ-S and TMZ-R cells showed that a significantly higher level of SNHG15, coincidental with a higher level of Sox2, β-catenin, EGFR, and CDK6 in TMZ-R cells. Subsequently, using bioinformatics tool, a potential mechanistic route for SNHG15 to promote GBM tumorigenesis was by inhibiting tumor suppressor, miR-627-5p which leads to activation of CDK6. Gene-silencing technique was employed to demonstrate that suppression of SNHG15 indeed led to the suppression of GBM tumorigenesis, accompanied by an increase miR-627-5p and decreased its two oncogenic targets, CDK6 and SOX-2. In addition, SNHG15-silenced TMZ-R cells became significantly sensitive towards TMZ treatment and less capable of promoting M2-phenotype in the HMC3 microglial cells. We then evaluated the potential anti-GBM activity of CDK6 inhibitor, palbociclib, using TMZ-R PDX mouse models. Palbociclib treatment significantly reduced tumorigenesis in TMZ-R/HMC3 bearing mice and SNHG15 and CDK6 expression was significantly reduced while miR-627-5p level was increased. Additionally, palbociclib treatment appeared to overcome TMZ resistance as well as reduced M2 markers in HMC3 cells.

**Conclusion:**

Together, we provided evidence supporting the usage of CDK6 inhibitor for TMZ-resistant GBM cases. Further investigation is warranted for the consideration of clinical trials.

**Graphical abstract:**

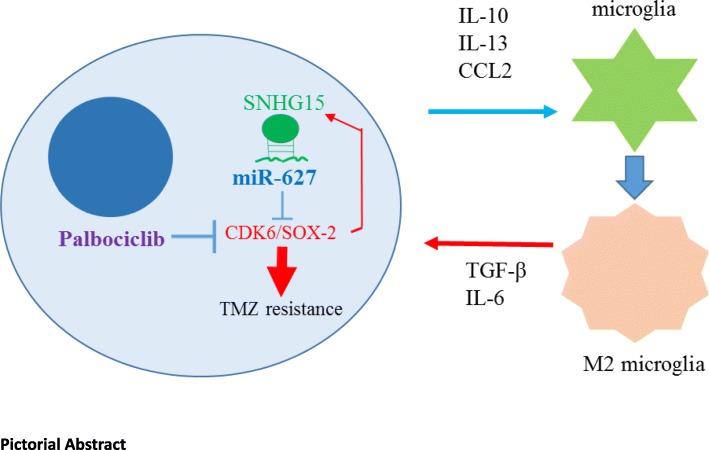

**Electronic supplementary material:**

The online version of this article (10.1186/s13046-019-1371-0) contains supplementary material, which is available to authorized users.

## Background

Glioblastoma (GBM) is one of the most lethal brain tumors. The average survival is 12–18 months and the 5-year overall survival is approximately 5% [1]. Even with aggressive interventions including surgery, or combination of radiotherapy and chemotherapies, the prognosis for the patients remains poor. One of the major underlying contributing factors to GBM’s malignancy is the resistance to both traditional and targeted therapies. Due to the inherent heterogeneous nature of GBM, the signaling pathways involved in the acquired drug resistance represents a complicated task to decipher. The role of tumor microenvironment adds another layer of complexity. Glioma-associated microglial cells (GAMs) are functionally similar to that of tumor-associated macrophages in the peripheral system and interact with GBM cells intimately via intracellular communications [2, 3]. GAMs have been found to secrete a spectrum of cytokines and signaling molecules to promote tumor proliferation, anti-apoptosis and angiogenesis/metastasis [4, 5]. Thus, a better understanding of the intracellular molecular communications between GBM and GAMs will provide foundation for therapeutic development.

Emerging evidence indicates the importance of abundantly transcribed non-coding genes termed long non-coding RNAs (lncRNAs) in virtually every aspect of cell biology including glioma tumorigenesis. Recently, a lncRNA, termed SNHG15 has gained much attention. SNHG15 was shown to be elevated and associated with tumorigenic functions including proliferation and metastasis in a variety of cancer types including breast, lung, and liver [6–8]. Notably, lncRNA SNHG15 level was positively linked to the histological grade, tumor node metastasis stage (TNM) and the poor overall survival in hepatocellular carcinoma [8]. However, the potential role of SNHG15 in GBM tumorigenesis has not been fully investigated. Initially, we searched public databases and identified that an elevated level of lncSNHG15 in GBM cells as compared to normal brain tissues, and this elevation of lncSNHG15 was associated with a significantly higher risk of developing GBM and a shorter survival time in the patients.

In this study, we employed in vitro and in vivo assays to demonstrate the tumorigenic roles of lncSNHG15. First, we found that a significantly higher level of lncSNHG15 in TMZ-resistant clinical GBM samples and was associated with GBM malignant properties. Increased lncSNHG15 level was associated with an increased expression in markers of oncogenesis such as EGFR, CDK6 and stemness including Sox2 and β-catenin. More importantly, TMZ-resistant (TMZ-R) GBM cells were more capable of promoting M2-polarization of glioma associated microglia (M2-GAMs) than TMZ-sensitive (TMZ-S) counterparts. Down-regulation of lncSNHG15 resulted in reduced tumorigenesis, self-renewal and increased TMZ sensitivity and the reverse was true when increased. Interestingly, CDK6 inhibitor, palbociclib treatment suppressed GBM tumorigenesis as well as the generation of M2 GAMs. Palbociclib’s anti-GBM effects were associated with a reduced level of lncSNHG15 and increased level of tumor suppressor miR-627. Mechanistically, miR-627 could target not only CDK6 but also Sox2 and β-catenin. Finally, we used patient-derived xenograft model to demonstrate that palbociclib treatment alone significantly reduced GBM tumorigenesis and with a greater extent when combined with TMZ.

In summary, we provided preclinical insights into the functional roles of lncSNHG15/CDK6/miR-627 regulatory circuit in the development of GBM and polarization of GAMs. More importantly, the feasibility of employing palbociclib for treating TMZ-resistant GBM was examined and supported by both in vitro and in vivo models.

## Materials and methods

### Ethics approval and consent to participate

Clinical samples were collected from Harbin Medical University (Harbin, China). All enrolled patients gave written informed consent for their tissues to be used for scientific research. The study was approved by the Institutional Review Board (IRB) of the Harbin Medical University (Harbin, China), consistent with the recommendations of the declaration of Helsinki for biomedical research (Harbin Medical University, Harbin, China) and followed standard institutional protocol for human research. Moreover, the animal study protocol was approved by the Animal Care and User Committee at Harbin Medical University (Harbin, China) (Affidavit of Approval of Animal Use Protocol # Harbin Medical University).

### Cell culture and clinical sample collection

Forty cancer tissues from the patients diagnosed with glioma (with different grades, please refer to Additional file 1: Table S1 for clinicalpathological features) were collected for this study. All tissue samples were pathologically confirmed and immediately snap-frozen in liquid nitrogen until RNA extraction. Written informed consent to the use of the tissue samples for research purposes was obtained from each patient. All procedures were conducted in accordance with the principles outlined in the Declaration of Helsinki, and all applicable international, national and/or institutional guidelines for the care and use of animals were followed. The study protocol was approved by the Ethics Committee of Harbin Medical University (Harbin, China). TMZ-resistant (termed TMZ-R) and TMZ-sensitive (termed TMZ-S) clinical samples were collected and cultured for further analyses in this study. The procedures used to isolate and culture TMZ-R and TMZ-S cells were according to a previously published study [9]. The human microglial cell line, HMC3 used in our study was obtained from American Type Culture Collection (ATCC, Manassas, VA, USA). HMC3 cells were cultured according to the recommendations by ATCC, where EMEM (ATCC® 30–2003™) was used as the base medium and completed by adding 56 mL FBS (ATCC® 30–2020™) to a 500 mL of base EMEM.

### Co-culture and GAM polarization

Co-culture assays of tumor cells with macrophages TMZ-R and TMZ-S GBM cells were co-cultured with HMC3 microglia (using Boyden Chamber) at a density of 1:10 or 1:5 in a 6-well plate. After 72 h, HMC3 were analyzed for their M1, M2 phenotypes using real-time PCR technique. This system was then used for testing palbociblic’s influence on GAM polarization, palbociblic (0.5 μM) was added into the TMZ-R and HMC3 co-culture system after the seeding and cultured for 72 h. The same experimental conditions were used for co-culturing lncSNHG15-silenced or overexpressed TMZ-R cells with HMC3 cells. Primer sequences for M1 M2 markers can be found in Additional file 2: Table S2. M1 M2 cytokines secreted into the culture medium were determined using M1/M2/MDSC Cytokines, ELISA Kit (Cat# MBS590066, MyBiosources.com). The procedures were performed according to vendor’s protocols.

### Total RNA isolation and qRT-PCR analyses

The isolation of total RNA and qRT-PCR were carried out as previously described [10]. All experiments were repeated in triplicates. Please refer to Additional file 2: Table S2 for primer sequences used in this study.

### Gene-silence and overexpression experiments

For gene silencing experiments, shRNA (Santa Cruz Biotechnology, USA) was constructed with sequences specifically against lncSNHG15. miR-627-5p mimic and inhibitor molecules and negative controls (NC) were purchased from GenePharma (Shanghai, China). For overexpression experiments, pcDNA3.1 (+) vector (GenePharma, Shanghai, China) was obtained to construct a pcDNA3.1-SNHG15 overexpressing plasmid. Please refer to Additional file 2: Table S2 for siRNA sequences**.**

### SDS-PAGE and Western blots

Expression analyses in this study were all carried out sing standard SDS-PAGE (10–12%). Cellular protein lysates were subsequently transferred to nitrocellulose membranes (Sigma), washed and incubated with primary antibodies in cold overnight, followed by washes and secondary antibodies incubation. Primary antibodies used were all purchased from Cell Signaling Technology unless otherwise specified. Please refer to Additional file 3: Figure S1. Full-size blots of Fig. 2e. Figure S2. Full-size blos of Fig. 4b. Figure S3. Full-size blots of Fig. 6a. 

### Colony formation assay

The colony formation assay was performed to determine the proliferation and evaluation the effects of gene manipulation and/or drug treatment of GBM cells. Briefly, GBM cells (500 cells per well) were incubated in 6-well plates. One week to 10 days, formed colonies were fixed with methanol and stained with 0.1% crystal violet. Colonies were quantified under a light microscope (Olympus Corp.). The experiments were done in triplicates.

### MTT assay

Cell viability assay or (MTT assay) was used to determine the viability, drug and/or gene manipulation effects on the GBM cells. GBM cells (5000 cells per well) were seeded in 96-well plates. Control and cells with different treatment were incubated with 20 μL MTT (5 mg/mL) in each well at the indicated time points (cells were collected at 0, 24, and 48 h). DMSO (Sigma) was added (150 μL/well) to each well to dissolve the crystals. OD was read at 490 nm on a microplate reader (Molecular Devices, Sunnyvale, CA, USA). The experiments were carried out in triplicates.

### Preclinical mouse model for drug evaluation

Immune compromised NOD/SCID females (6 weeks old) were supplied by Animal Care facility of the Harbin Medical University (Harbin, China). The animal study protocol was approved by the Animal Care and User Committee at Harbin Medical University (Harbin, China) (Affidavit of Approval of Animal Use Protocol # Harbin Medical University). Patient-derived TMZ-R cells (500,000 cells per injection) were injected subcutaneously. Mice were monitored weekly for tumor development using a standard caliper. Mice were subdivided into 4 groups, vehicle control, TMZ only (4 mg/kg, p.o., 5 times a week), palbociclib only (75 mg/kg, i.p injection, 5 times a week) and combination. At the end of experiments, mice were humanely euthanized or when tumor burden became symptomatic. Tumor samples were harvested for further analyses. Tumor growth were calculated using the following formula where tumor volume = (a × b^2^)/2, where a is the long axis and b is the short axis of the tumor.

### Statistical analysis

SPSS software (version 22.0, USA) was used to perform statistical analyses. The significant difference between different groups was analyzed using t-test or a chi-square test. The level of *P* value ≤0.05 is considered statistically significant.

## Results

### Increased lncSNHG15 level is associated with a poor prognosis in patients of GBM

**Fig. 1:**
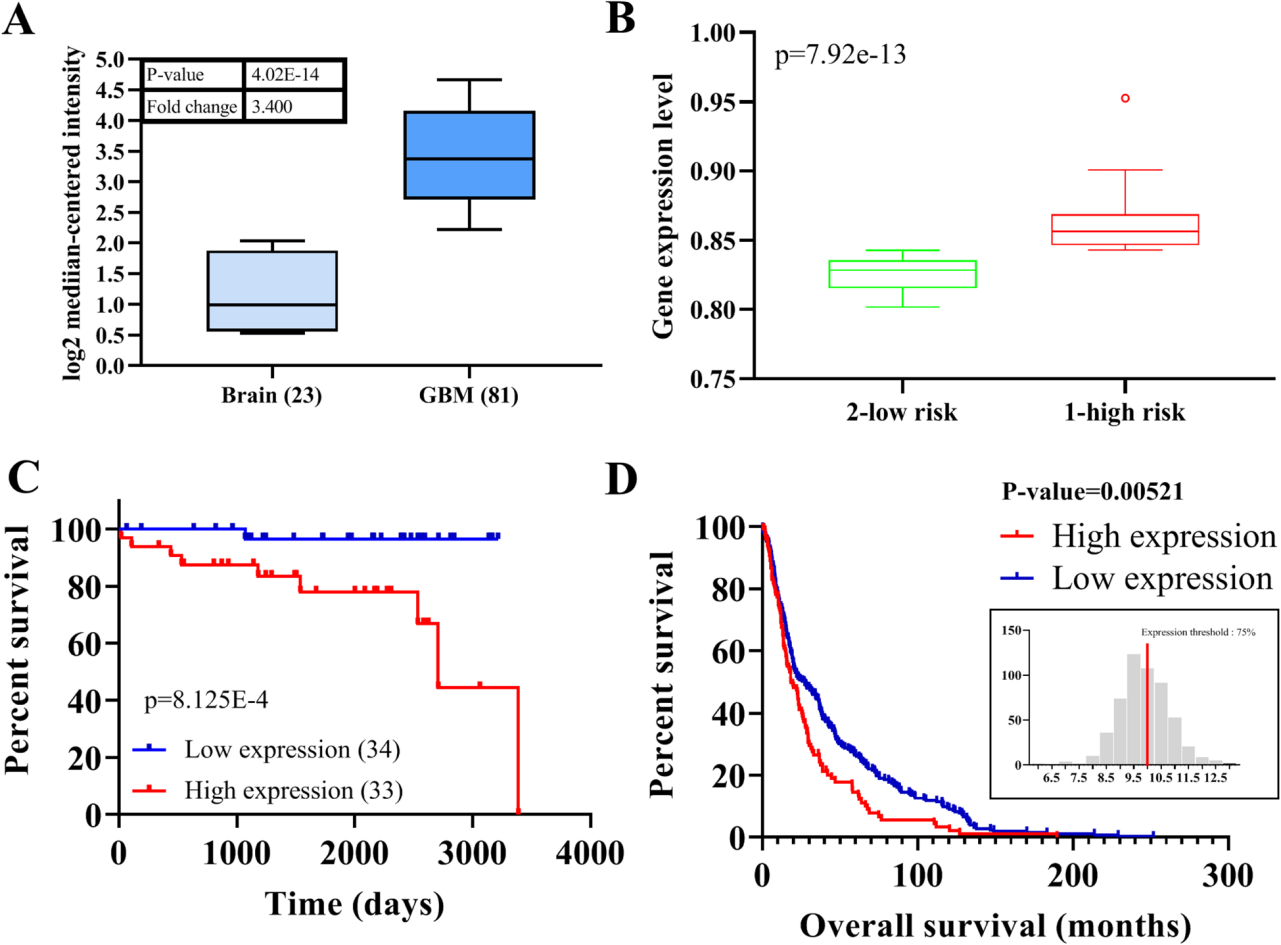
Increased lncSNHG15 in GBM cells and is associated with a poor prognosis. **a** Analysis of GBM database (GSE4290) showed that the level of lncSNHG15 was significantly higher in the GBM clinical samples as compared to the normal brain. Numbers in parentheses indicate the sample number. **b** In another database (GSE16581), a higher level lncSNHG15 was also identified in the patients with a higher risk for GBM. **c** Kaplan-Meier survival curve was drawn from the same cohort (GSE16581) and showed a higher level of lncSNHG15 was significantly associated with a lower survival ratio. The number of patients in each arm is indicated. **d** Using another database (GSE108476), a higher lncSNHG15 level statistically predicted a shorter survival time in the patients with glioma (*N* = 524)

**Fig. 2:**
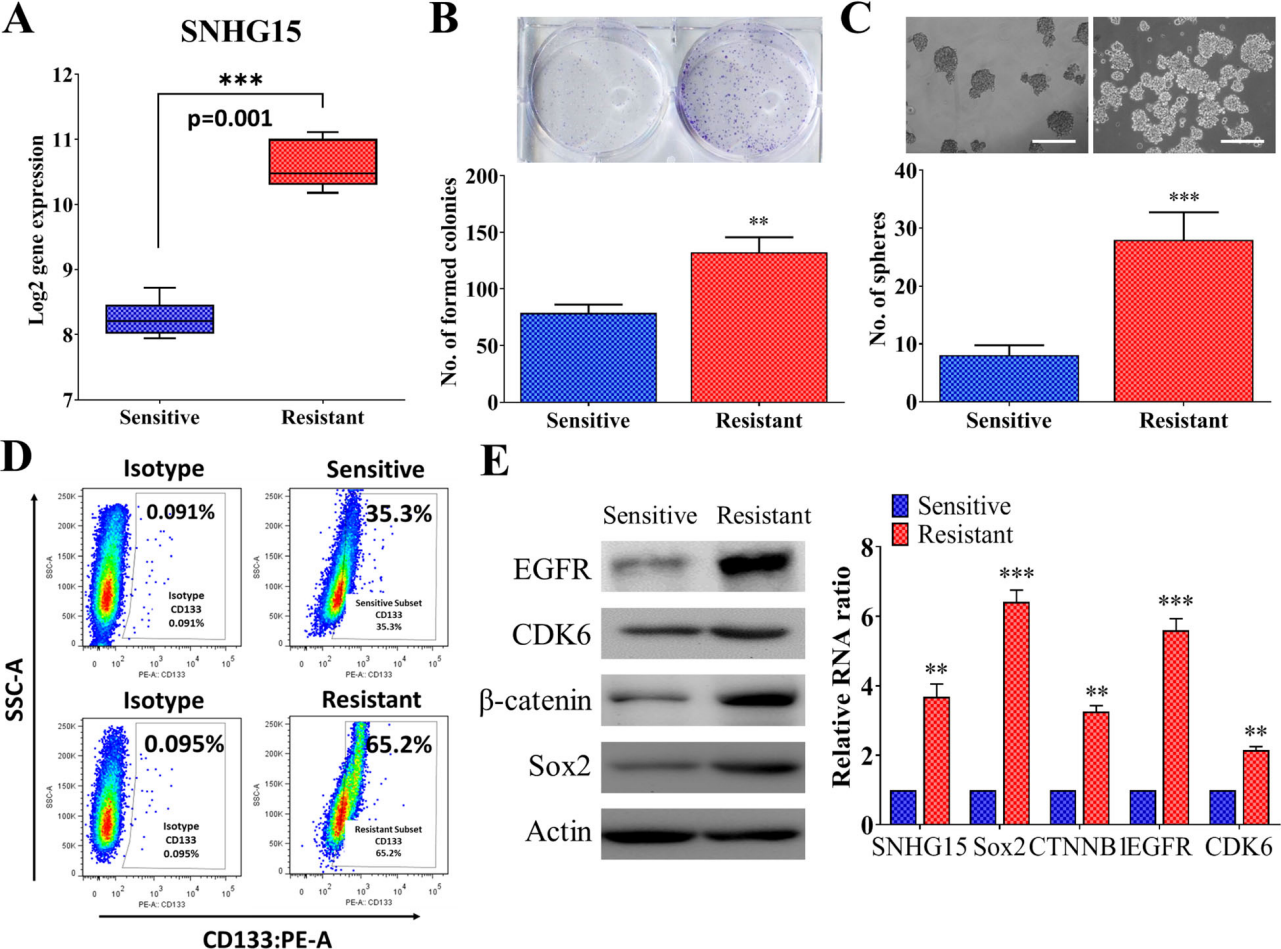
A higher level of lncSNHG15 in TMZ resistant GBM cells and associated with GBM malignancy (**a**) Comparative lncSNHG15 expression between TMZ resistant and sensitive clinical GBM samples. Real-time PCR analysis showed that a significantly higher level of lncSNGH15 in samples from patients who are non-responsive towards TMZ as compared to ones with responsiveness. TMZ-resistant cells showed an enhanced colony (**b**) and neurosphere (**c**) forming ability over TMZ-sensitive counterparts. **d** Comparative flow cytometric analysis also showed that TMZ-R cells contained a significantly higher percentages of CD133+ population (approximately 30% more) than their TMZ-S counterparts. **e** Comparative qPCR and western blot analysis of TMZ-resistant and TMZ-sensitive GBM cells. The lncSNHG15 was significantly higher in the TMZ-R cells as compared to TMZ-S cells. Also, the expression of stemness markers, Sox2, β-catenin and oncogenic markers EGFR and CDK6 were significantly lower in the TMZ-sensitive cells as compared to its TMZ-resistant counterpart. Neural differentiation marker GFAP was slightly higher in the TMZ-sensitive GBM cells

Previously, increased level of lncSNHG15 has been linked to malignant characteristics of cancer cells [11–13] but its role in GBM has not been fully appreciated. Here, we first compared the level of lncSNHG15 between normal brain tissue and GBM (database GSE4290) [14] and found that lncSNHG15 was significantly higher in the GBM samples (approximately 3.4 fold higher) as compared to that in the normal brain (Fig. 1a). In addition, analysis performed on another database (GSE16581) [15] showed that a higher expression of lncSNHG15 is associated with a high risk for GBM (Fig. 2b). Kaplan-Meier survival curve of the same cohort indicated that patients with a higher lncSNHG15 expression had a shorter survival time as compared to ones with a lower level of lncSNHG15 (Fig. 1c). Moreover, we examined a larger cohort of patients with glioma (GSE108476, *N* = 524) [16], a similar observation was made where patients with a higher lncSNHG15 was predicted to have a significantly shorter survival time (Fig. 1d). These observations strongly suggest that an increased level of lncSNHG15 plays an important role in GBM tumorigenesis.

### LncSNHG15 is elevated in the TMZ-resistant GBM cells and associated with stemness

Next, we examined the amount of lncSNHG15 from 5 pairs of non-tumors versus GBM grade 4 clinical samples, previously collected from our neurosurgical department. Comparatively, the level of lncSNHG15 was found significantly higher in the TMZ-resistant samples as compared to their TMZ-sensitive counterparts (Fig. 2a). Comparatively, TMZ-resistant GBM cells formed a significantly higher number of colonies as compared to their TMZ-sensitive counterparts (Fig. 2b). Subsequently, we cultured both TMZ resistant and sensitive cells under serum-derived conditions to examine the neurosphere-generating ability. TMZ-resistant cells formed a significantly higher number of neurospheres as compared to the sensitive counterparts (Fig. 2c). Data from flow cytometric analysis supported this observation where TMZ-resistant GBM samples contained a significantly higher percentage of CD133+ cells as compared to their TMZ-sensitive counterparts (Fig. 2d). In support, comparative expression analysis revealed TMZ-resistant GBM cells contained a higher level of stemness markers including Sox2, β-catenin and oncogenic markers, EGFR and CDK6 (Fig. 2e) in both Western blot and q-RT-PCR detection.

### TMZ-resistant GBM cells promote the generation of M2 glioma-associated microglial cells (GAMs) and tumorigenic properties

**Fig. 3:**
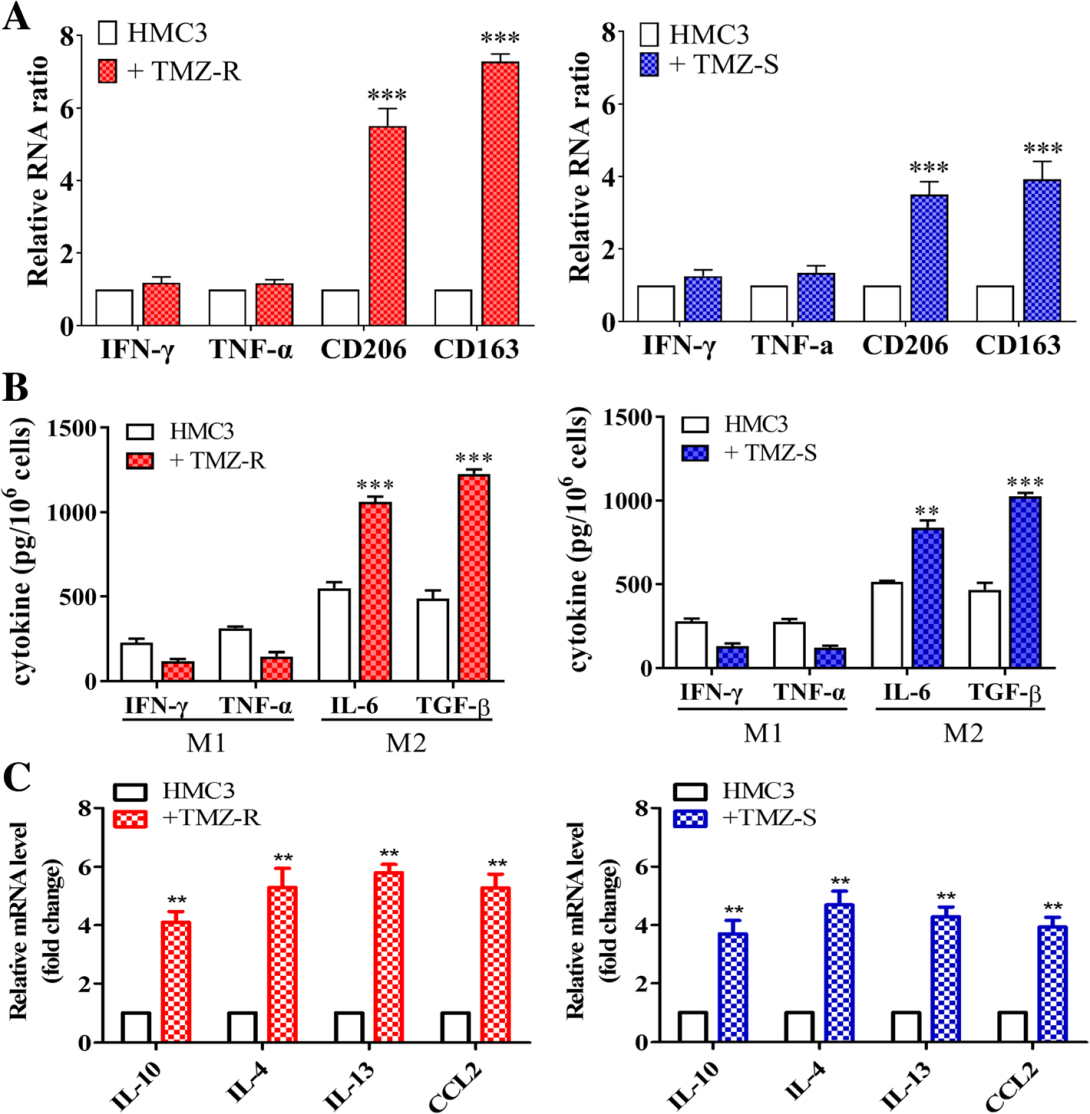
TMZ-resistant GBM cells promotes generation of M2 GAMs. **a** Real-time PCR analysis showed TMZ-R GBM co-cultured HMC3 (microglial cells) showed a significantly increased level of M2 markers CD206 and CD163 but not M1 markers INF-γ and TNF-α; similar observations were made in TMZ-S co-culture but with lower elevation of M2 markers. **b** ELISA assays showing the increased M2 cytokines secreted by co-culturing TMZ-R and TMZ-S GBM cells with HMC3 microglial cells. TMZ-R co-cultured HMC3 microglia released an increased level of M2 cytokines, TGF-β and IL-6 but decreased M1 cytokines, INF-Ƴ and TNF-α; TMZ-R co-cultured HMC3 showed similar results but with lower level of M2 cytokines as compared to TMZ-R counterparts. **c** Real-time PCR analysis demonstrated the increased post TMZ-R with HMC3 microglia co-cultured. An increased mRNA level of IL-10, IL-4, IL-13 and CCL2 was observed. **p < 0.01; ***p < 0.001

We collected and cultured TMZ-resistant (TMZ-R) and TMZ-sensitive (TMZ-S) from patients (please refer to Additional file 1: Table S1 for clinicopathological information) and found that TMZ-R cells significantly induced the M2 markers, CD163 and CD206 in GAMs; TMZ-S cell also promoted M2 polarization in HMC3 cells but with a less extent as compared to TMZ-R cells (Fig. 3a); the level of M1 markers, INF-γ and TNF-α were slightly decreased but not statistically significant. Next, we examined and compared the secreted M2 cytokines in GAMs in the presence of TMZ-R and TMZ-S. In accordance, M2 cytokines, IL-6 and TGF-β secreted by GAMs co-cultured with TMZ-R were higher than naïve GAM counterparts (Fig. 3b). For instance, IL-6 and TGF-β level was approximately 2-fold and 3-fold respectively higher in the TMZ-R co-cultured GAMs (left panel, Fig. 3b). A similar observation was made in the GAMs co-cultured with TMZ-S cells but with lesser extent (right panel, Fig. 3b). On the front of cancer cells, both TMZ-R and TMZ-S cells showed a significant increase in the M2 cytokines mRNA level of IL-10, IL-4, IL-13 and CCL2 as compared to their parental counterparts (Fig. 3c).

### SNHG15 silencing led to decreased GBM tumorigenesis and increased TMZ sensitivity

**Fig. 4:**
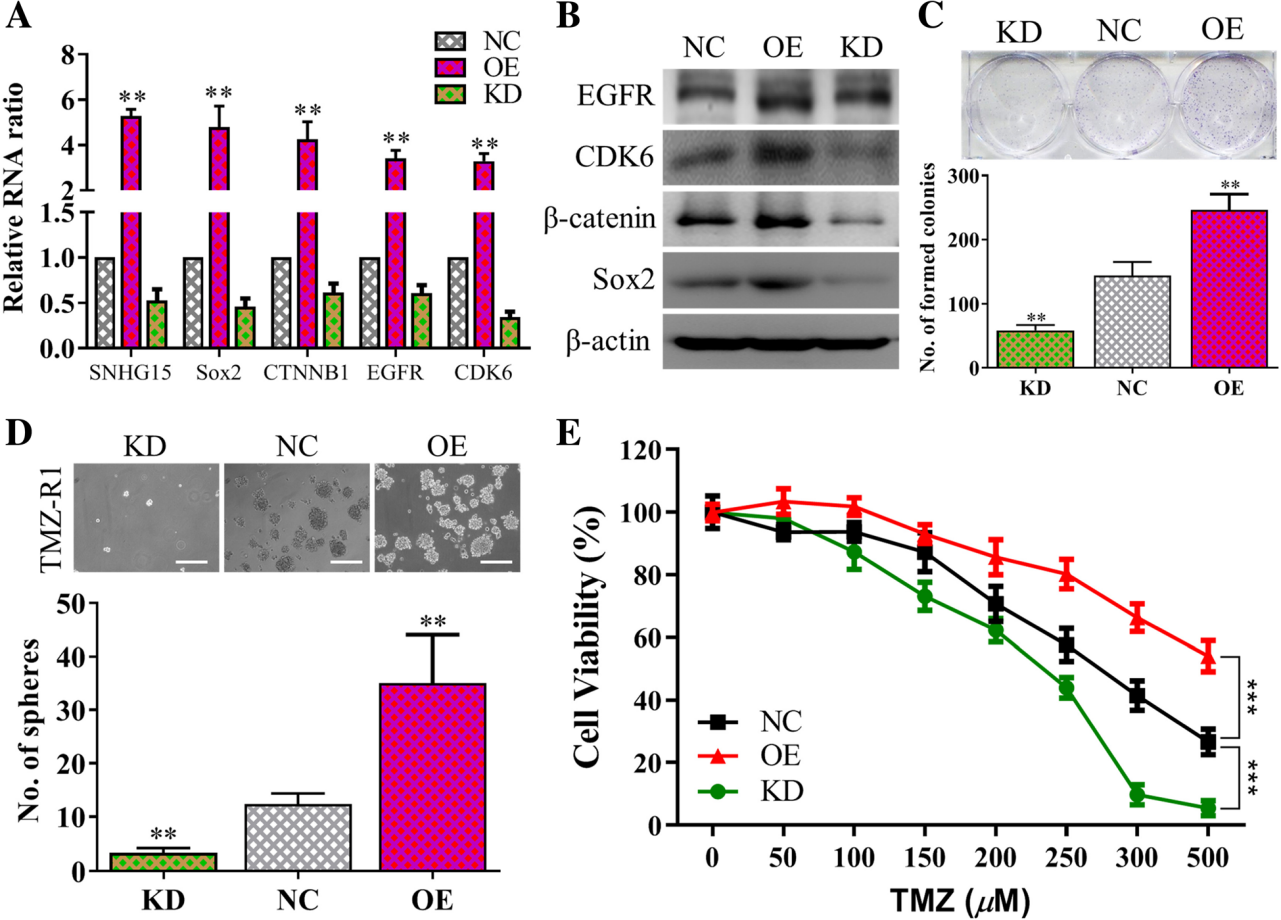
IncSNHG15 is associated with GBM malignancy and drug resistance. **a** Real-time PCR (**a**) and western blot (**b**) analyses of TMZ-R cells with lncSNHG15 silenced (KD) or overexpressed (OE). Expression level of lncSNHG15 is associated with oncogenic markers, CDK6, Sox2, β-catentin and EGFR as demonstrated by overexpressing lncSNHG15 (OE) and reduced expression of these markers was found in the lncSNHG15-knocked down (KD) TMZ-R cells. LncSNGH15-silenced TMZ-R cells appeared to form a significantly lower number of colonies (**c**) and nerospheres (**d**) as compared to NC and OE counterparts. **P < 0.01; ***P < 0.005. **e** Drug sensitivity assay. LncSNHG15-silenced TMZ-R cells became more sensitive (IC50, 225 μM) towards TMZ treatment while lncSNHG15-overexpresed TMZ-R became even more resistant (IC50 > 500 μM) against TMZ, as compared to NC control (IC50, 310 μM)

To determine the functional connection between lncSNHG15 and GBM tumorigenesis, gene-silencing experiment was performed. First, expression profiles were performed and showed that post lncSNHG15 silencing, stemness markers including Sox2, β-catenin, and oncogenic markers EGFR and CDK6 were significantly reduced (qPCR analysis, left panel; western blots, right panel, Fig. 4a). In support, western blots of TMZ-R cells with lncSNHG15 silenced (KD) or overexpressed (OE) demonstrated that expression of lncSNHG15 was positively associated with the expression of oncogenic markers including Sox2, β-catenin, EGFR and CDK6 (Fig. 4b). Next, SNHG15 knockdowned GBM cells (TMZ-R1 KD cells) showed a significantly lower ability to form colonies and neurospheres as compared to its parental counterpart (Fig. 4c) whereas opposite observations were made in TMZ-R cells overexpressing lncSNHG15 (Fig. 4d). More importantly, lncSNHG15 level was positively correlated with TMZ sensitivity. For instance, silencing lncSNHG15 led to an increased TMZ sensitivity in TMZ-R cells as compared to its parental counterpart (Fig. 4e), as the IC_50_ value decreased from approximately 310 μM to 225 μM post lncSNHG15 silencing and increased to more than 550 μM in the lncSNHG15-overexpressing cells (Fig. 4e).

### Palbociclib treatment suppressed GBM tumorigenesis was associated with downregulation of lncSNHG15

**Fig. 5:**
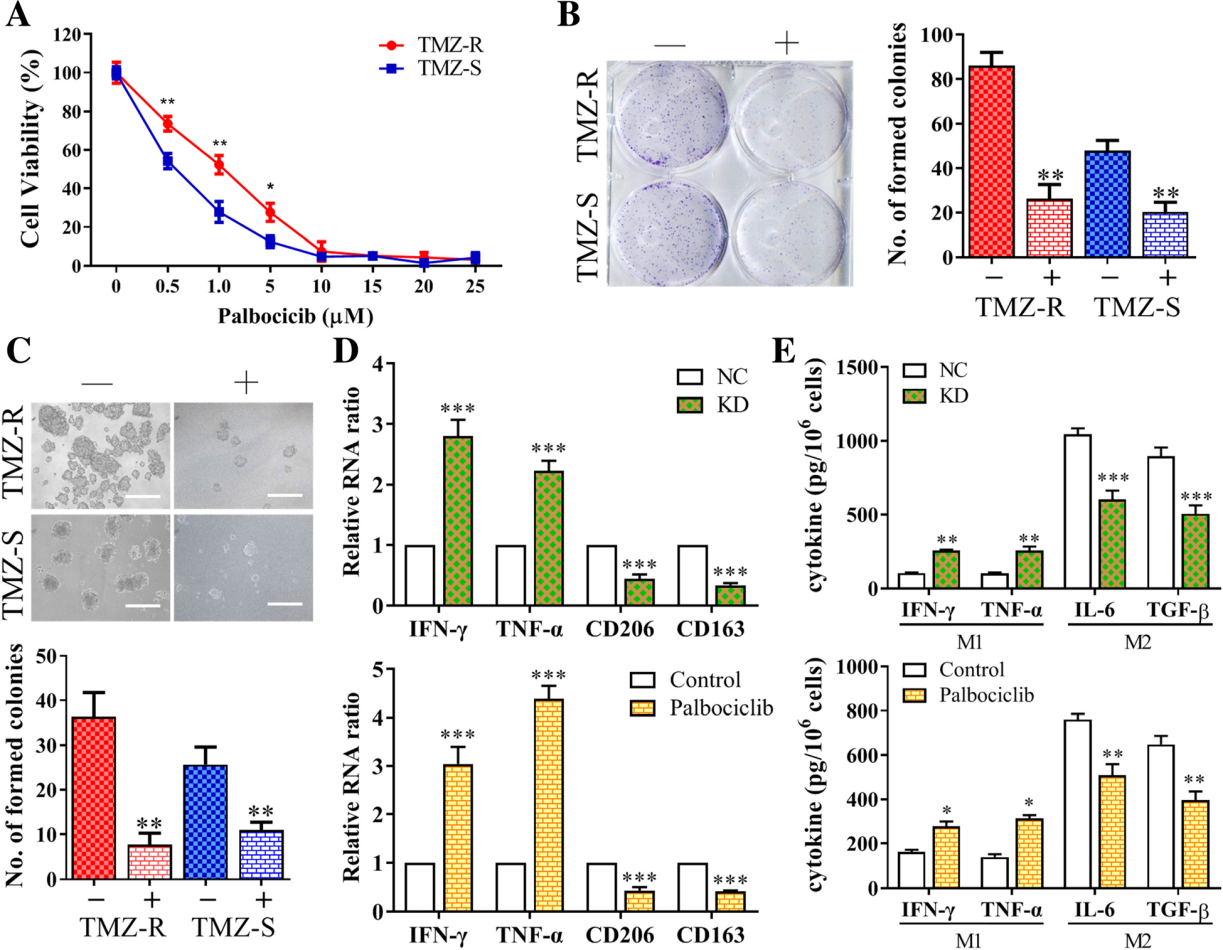
Palbociclib treatment suppressed GBM tumorigenesis and M2 polarization. **a** Cell viability assay of palbociclib in TMZ-R and TMZ-S cells. Palbociclib exerted a potent inhibitory effect on cell viability in both TMZ-R and TMZ-S cells with estimated IC50 values of 1.0 μM and 0.5 μM respectively. Palbociclib treatment significantly reduced the colony (**b**) and neurosphere (**c**) forming ability in both TMZ-R and TMZ-S cells. **d** Real-time PCR analysis showed that lncSNHG15-silencing (upper panel) and palbociclib treatment (bottom panel) rendered TMZ-R cells incapable of promoting M2 polarization in macrophages. M1 markers, IFN-γ and TNF-α, were increased while M2 markers CD163 and CD206 were decreased. **e** Similarly, co-culturing TMZ-R cells with lncSNHG15-silenced (upper panel) or treated with palbociclib (bottom panel) decreased M2 cytokines (IL-6 and TGF-β) and increased M1 cytokines (IFN-γ and TNF-α) secreted by the GAMs

CDK6 has been shown to be overexpressed in GBM and according to our data, CDK6 level is higher in the TMZ-R GBM cells. Here, we test the potential of using CDK6 inhibitor, palbociclib for the treatment of TMZ resistant GBM cells and associated effects on the tumor microenvironment. First, we showed that palbociclib treatment significantly suppressed the proliferation of TMZ-R and TMZ-S cells (Fig. 5a). We also observed palbociclib treatment significantly suppressed GBM cells to form colonies (Fig. 5b) and neurospheres (Fig. 5c). Equally important, we examined the effects of lncSNHG15 silencing and CDK6 inhibition on TAM polarization. LncSNHG15-silenced TMZ-R cells showed a significantly decreased ability to generate M2 GAMs and appeared to promote M1 polarization, as demonstrated by the increased M1 markers IFN-γ and TNF-α while decreased M2 markers CD163 and CD206 (left panel, Fig. 5d); the M1 cytokines, IFN-γ and TNF-α were also increased in the GAMs co-cultured with lncSNHG15-silenced TMZ-R cells while M2 cytokines, IL-6 and TGF-β were prominently suppressed (left panel, Fig. 5e). Consistently, palbociclib treatment resulted in similar observations where M1 markers and cytokines were upregulated while M2 suppressed (right panel, Fig. 5d and right panel, 5E).

### Palbociclib suppressed GMB tumorigenesis via down-regulation of CDK6 and was associated with the upregulation of tumor suppressor miR-627

**Fig. 6:**
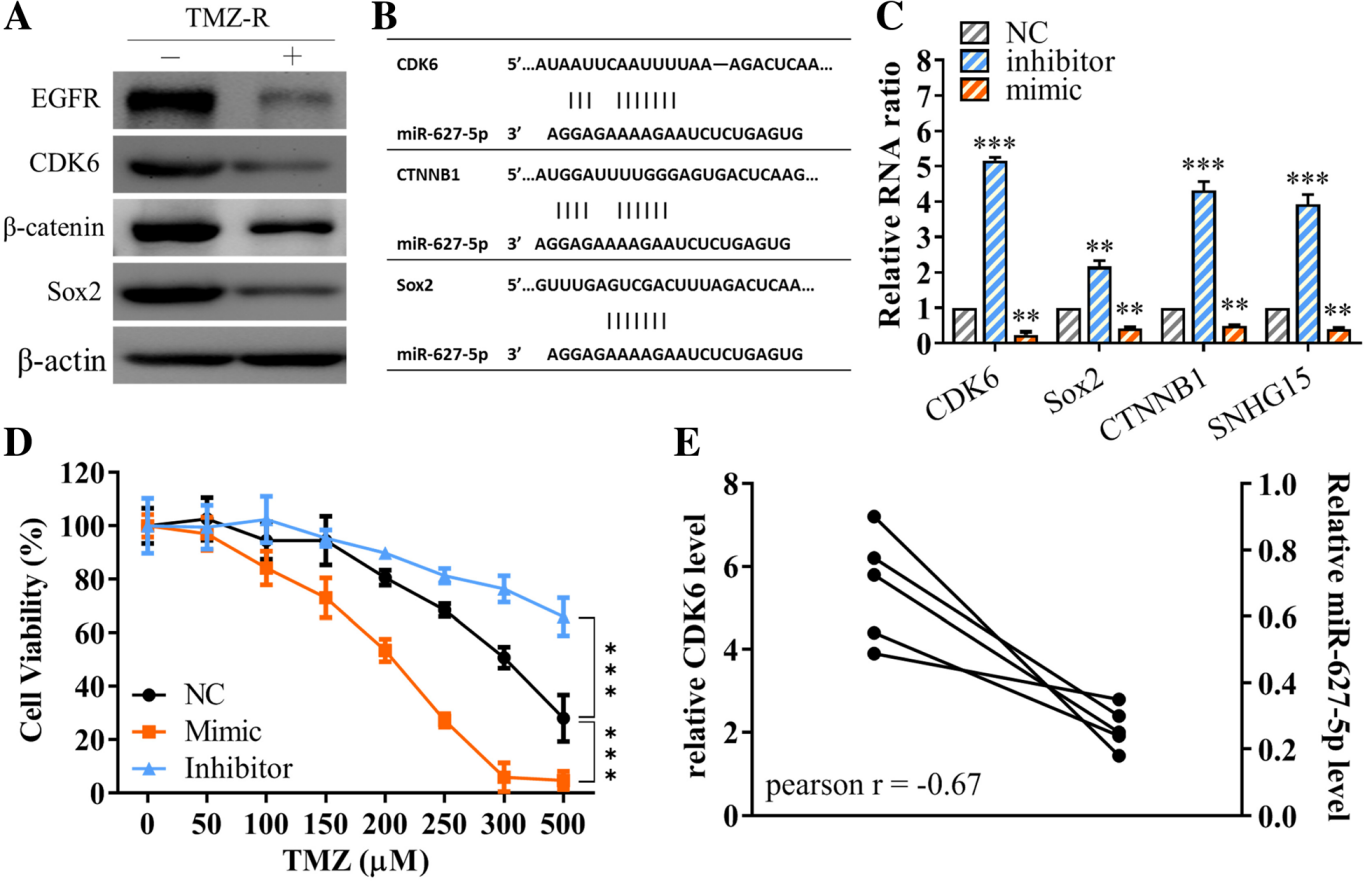
Palbociclib mediated GMB suppression is associated with an increased in miR-627-5p. **a** Western blots of TMZ-R cells without (−) and with (+) the treatment of palbociclib. Post palbociclib treatment, the expression of CDK6, EGFR, Sox2 and β-catenin was prominently lower as compared to their un-treated counterparts. **b** Predicted binding sites of miR-627-5p’s targets. Different algorithms were used to predict the potential targets for miR-627-5p such as CDK6, CTNNB1 and Sox2. **c** Real-time PCR analysis showed that inhibitor of miR-627-5p transfection led to increased level of CDK6, Sox2, CTNNB1 and lncSNHG15 while the opposite was observed in TMZ-R cells transfected with miR-627-5p mimic. **P < 0.01; ***P < 0.005. **d** TMZ sensitivity was associated with the level of miR-627-5p. TMZ-R cells with exogenous miR-627-5p (mimic) became more sensitive (approximately 200 μM, 48 h) towards TMZ as compared to the NC (approximately 300 μM, 48 h) whereas decreased miR-627-5p by inhibitor led to a significantly increased resistance against TMZ (IC50 > 500 μM, 48 h). **e** Negative correlation between CDK6 and miR-627-5p level in TMZ-resistant clinical samples

Western blots of TMZ-R cells treated with palbociclib showed the decreased expression of oncogenic markers including CDK6, β-catenin, EGFR, and stemness marker, Sox2 (Fig. 6a). To further explore the regulatory signaling pathway(s) associated with CDK6, we employed 3 different algorithms (miRmap, PITA and microT) for predicting the microRNAs with the potential to target CDK6. We identified miR-627-5p could potentially bind to the 3′ untranslated region (3UTR) of CDK6 as well as Sox2 and β-catenin (Fig. 6b). We then overexpressed and silenced miR-627 to determine the consequential effect on CDK6 expression. TMZ-R cells transfected with miR-627-5p mimic molecules resulted in the significantly decreased CDK6 expression along with Sox2, β-catenin and lncSNHG15 (Fig. 6c). In addition, exogenous miR-627-5p (mimic molecules) resulted in the increased TMZ sensitivity of TMZ-R cells whereas inhibitor of miR-627-5p further increased TMZ resistance (Fig. 6d). For instance, the IC50 value of TMZ-R cells transfected with miR-627-5p mimic molecule was estimated 200 μM as compared to the NC’s (negative control) 300 μM and greater than 500 μM in the case of TMZ-R cells transfected with miR-627-5p inhibitor (Fig. 6d). In support, we observed a strong negative correlation (Pearson r = − 0.008) between CDK6 mRNA level and miR-627-5p in the clinical TMZ-R samples.

### Combination of palbociclib and TMZ suppresses TMZ-R tumorigenesis via downregulating CDK6 and M2 TAM infiltration

**Fig. 7:**
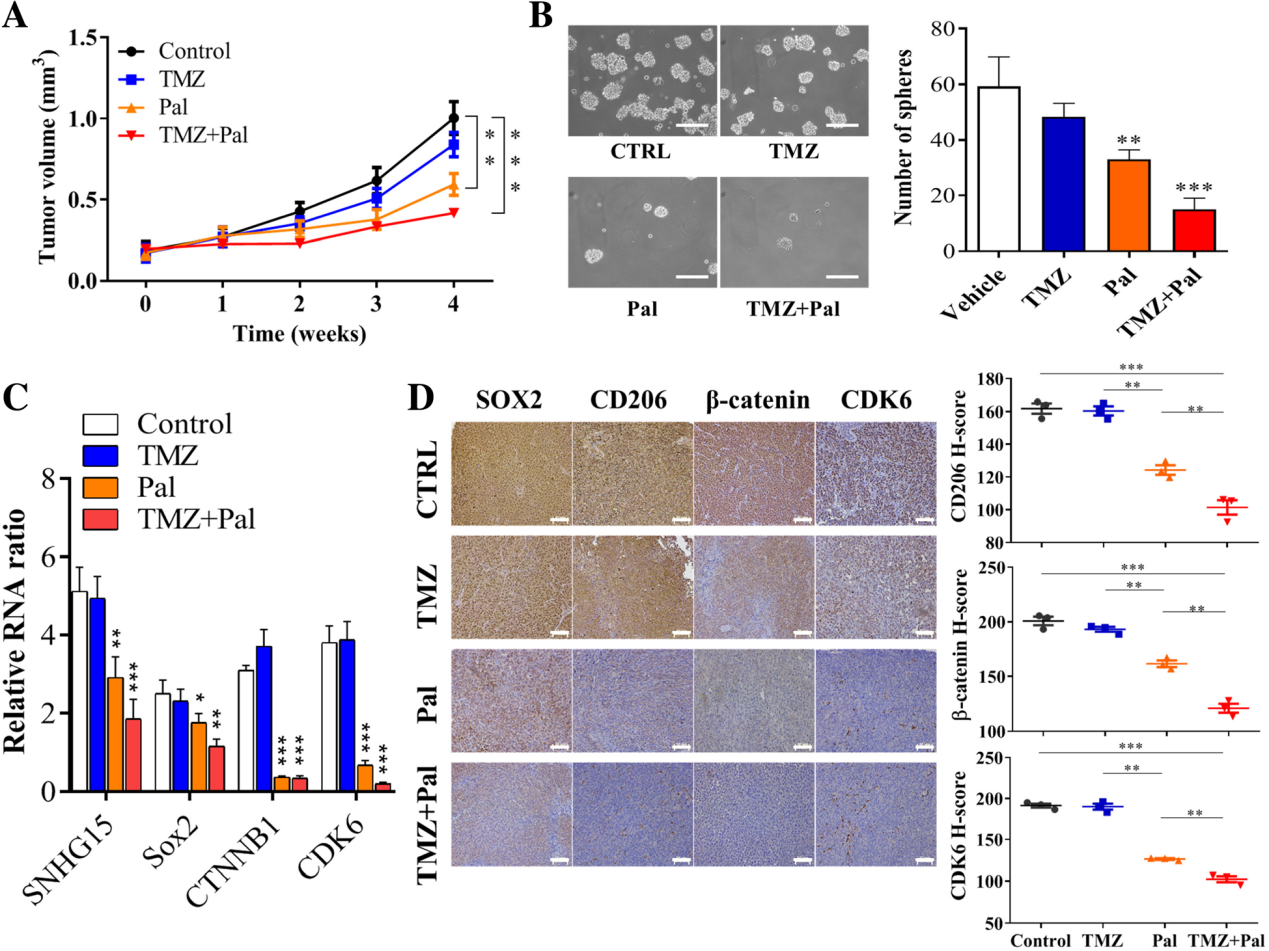
In vivo efficacy evaluation of the combination of TMZ and palbociclib in GBM PDX model. **a** Average tumor size versus time curves. The tumor growth was the most significantly delayed in the combination treatment group (TMZ and palbociclib) followed by palbociclib alone while TMZ alone and vehicle control group did not differ significantly. a, *P* < 0.01; b, P < 0.005. **b** Comparative neurosphere-generating ability. Tumor samples from each group were harvested and compared for their neurosphere-forming potential. The combination group showed the least number of neurospheres formed followed by palbociclib alone group, while TMZ and the control group showed similar degree of neurosphere-forming ability (highest). **c** Real-time PCR analysis. The combination group showed the lowest level of lncSNHG15, Sox2, CTNNB1, Sox2 and CDK6. a, *P* < 0.01; b, *P* < 0.005. **d** Immunohistochemical analysis of tumor samples. Comparatively, the immunohistochemical staining of CD206(M2 TAM marker), b-catenin and CDK6 was the lowest in the tumor samples collected from the combination group. The quantitative analysis of the IHC staining was represented by the H-score of each marker and represented by the dot-plot. **P < 0.01; ***P < 0.005

Finally, we examined the in vivo efficacy of palbociclib using TMZ-R patient-derived xenograft mouse model. We found that the tumor growth curve of TMZ-treated group did not significantly differ from the vehicle control group; mice treated with palbociclib alone showed significantly delayed tumor growth and the combination of TMZ and palbociclib yielded the most significantly delayed tumorigenesis in vivo (Fig. 7a). Tumor samples were harvested and compared for their ability in generating neurospheres. In accordance, the combination treatment resulted in in the least neurospheres generated followed by palbociclib alone group, while TMZ and vehicle control showed the highest number of neurospheres generated (Fig. 7b). In addition, comparative qPCR analysis of the tumor samples showed that the combination treatment resulted in the lowest level of CDK6, β-catenin, Sox2 and lncSNHG15 (Fig. 7c). The degree of M2 TAM polarization was also demonstrated in the immunohistochemical analysis of the tumor samples. Consistently, the combination treatment group showed the least M2 TAM staining (CD206) and the lowest expression of CDK6 and β-catenin (Fig. 7d). The IHC staining was quantified using H-score and was represented by the dot plots (Fig. 7d).

## Discussion

Glioblastoma multiforme (GBM) is one of the most malignant cancer types and currently there is no effective regiments for combating this deadly disease. Despite advancement in the development of chemotherapeutic agents including targeted therapeutic agents, the overall survival time often does not extend beyond 2 years post diagnosis. Recent experimental and clinical insights indicate the tumor microenvironment (TME) of GBM also play instrumental roles in promoting tumorigenesis and the development of drug resistance. Glioma-associated microglia (GAM) represents one of the key cell types that have been shown to contribute significantly towards GBM tumorigenesis and progression [17, 18]. In this study, we examined the potential signaling networks involved in promoting TMZ resistance and the feasibility of using CDK6 inhibitor, palbociclib as a candidate agent for treatment.

First, we found that an emerging tumorigenic lncRNA, SNHG15 is elevated in the GBM clinical samples and associated with a significantly shorter survival time (Fig. 1). We also provide clinical validation using our own clinical samples demonstrating a significantly higher lncSNH15 level in the TMZ-R cells than TMZ-S cells, implicating lncSNHG15’s potential involvement in drug resistance (Fig. 2). We then showed that TMZ-R cells with a higher level lncSNHG15 possessed a significantly higher self-renewal capacity, coincident with the higher expression of Sox2, β-catenin and EGFR, all of which have been attributed to GBM’s ability to fend off therapeutics [19–21]. In addition, our observation was supported by a recent study where lncSNHG15 level was found elevated in the endothelial cells and involved in promoting angiogenesis [22]. In addition, an increased level of lncSNHG15 was linked to the increased metastatic potential of non-small cell lung cancer [23] and liver metastasis in colon cancer [24]. Results from this study and others support the tumorigenic role of lncSNHG15.

Notably, we added another layer of complexity in terms of lncSNHG15’s role in GBM tumorigenesis where the increased lncSNH15 level was associated to the propensity to promote M2 polarization in the GAM. This association was supported by the observations where lncSNHG15 silencing significantly reduced M2 polarization while towards M1 polarization (Fig. 3). Moreover, we showed a positive correlation between the level of lncSNHG15 and oncogenic/stemness markers such as EGFR, CDK6, SOX-2 and β-catenin (Fig. 4). This observation added another possible target for drug development for treating GBM since these markers not only have been documented to be elevated in GBM but also contribute towards malignancy. For instance, β-catenin signaling was linked to the generation of CD133+ glioma stem cells [25] as well as linked to the increased oncogenic activity of EGFR [26]; more importantly, CDK6 has been shown to be an emerging target for drug development for GBM where CDK6 expression/activity is elevated in GBM cells and inhibitor of CDK6 could significantly suppressed GBM in vitro and in vivo [27]. Based on these studies and our observations, we have further supported the tumorigenic role of lncSNHG15 in GBM.

To further explore the connection between CDK6 and lncSNHG15, we showed that treating TMZ-R cells with CDK6 inhibitor, palbociclib also led to the decreased level of lncSNHG15; palbociclib-treated TMZ-R cells showed a significantly decreased ability to generate M2 GAM (Fig.5). Consistently, palbociblic treatment (suppression of CDK6) led to decreased expression of EGFR, Sox2 and β-catenin, similarly seen in the lncSNHG15-silenced TMZ-R cells. More importantly, lncSNHG15 knockdown and palbociclib treatment both led to an increased sensitivity towards TMZ. Mechanistically, we identified that a tumor suppressor, miR-627-5p targeted CDK6 at its 3’UTR and palbociclib treatment led to an increased level of this tumor suppressor (Fig. 6). Interestingly, in a previous report, miR-627 was found to target a histone demethylase (JMJD1A) and show anti-cancer function in colon cancer [28]. Since JMJD1A is an epigenetic regulator, it was logical that palbociclib-induced miR-627 level could suppress tumorigenesis via a myriad of targets. Finally, our PDX study showed that the addition of palbociblic could overcome TMZ-resistance in vivo (Fig. 7).

Palbociclib (trade name: Ibrancea, approved by FDA in February 2015 to treat HR+/HER2- breast cancer), amebaciclib and ribociclib (trade name: Kisqali, approved by FDA in March 2017 to treat HR+/HER2- breast cancer) are recently developed CDK4/6 inhibitors currently undergoing clinical testing as potential chemotherapeutics for the treatment of primary or secondary brain tumors [29]. Palbociclib was shown to promote survival in a genetic mouse model of brain stem glioma, but its unbound brain-to-plasma partition coefficient was only 5 min after intravenous administration of 1 mg/kg [30]. There is preliminary evidence and preclinical data that ribociclib, abemaciclib and palbociclib cross the brain–blood barrier with data suggesting that albemaciclib may be more efficient in crossing the blood–brain barrier compared to palbociclb. Several clinical trials are ongoing to evaluate CDK4/6 inhibitors in patients with brain metastases.

In a Phase I dose escalation safety study, palbociclib was examined in 3 weeks on 1 week off schedule in 41 patients with advanced malignancies. Therecommended phase II dose, at which neutropenia was the sole significant toxicity was determined to be 125 mg once daily. As dose limiting toxicities, a reversible neutropenia was identified. Grade 3 hematological toxicities included neutropenia (12%) and anemia (7%). Non-hematological toxicity was mild, including fatigue, nausea and diarrhea. There was a clear signal for clinical activity of the drug: Thirty-seven patients were evaluable for tumor response; 10 (27%) had stable disease for ≥4 cycles of whom six derived prolonged benefit (≥10 cycles) [31]. Beyond breast cancer, new indications for palbociclib are currently being evaluated in other malignancies such as sarcoma, pancreatic cancer, head & neck cancer, NSCLC, brain tumors, or even hematological malignancies.

## Conclusions

**Fig. 8:**
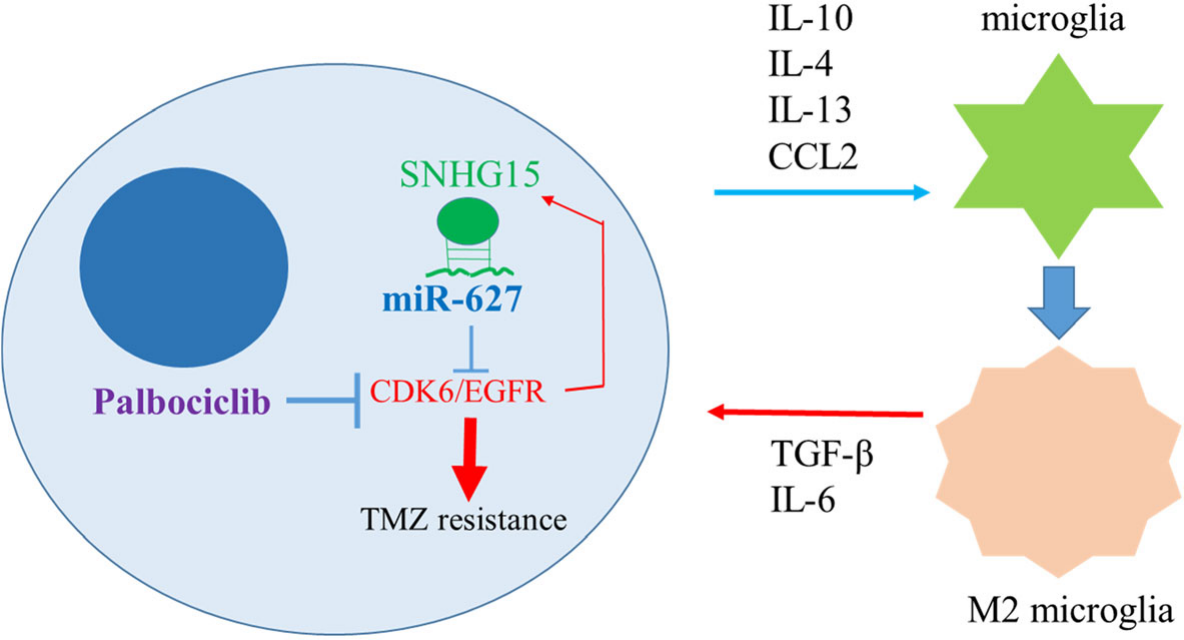
An increased level of lncRNA SNHG15 is associated with TMZ resistance by way of increasing CDK6/SOX-2 expression; in parallel, GBM promotes M2 polarization of microglia via secretion of pro-inflammatory cytokines, IL-10, IL-13, CCL2, etc. M2 microglial cells subsequently promotes GBM proliferation/survival via secretion of TGF-β and IL-6. Palbociclib inhibits CDK6 and could in turn decreases the level of SNHG15 and increases the tumor suppressor, miR-627-5p, leading to GBM vulnerable to TMZ treatment

In graphical summary (Fig. 8), we showed that the increased lncSNHG15 not only linked to the increased oncogenic and stemenss markers, Sox2, β-catenin, EGFR and CDK6 but also associated to GBM cells’ ability to promote M2-polarization of GAM. Using CDK6 inhibitor, palbociclib could effectively suppress tumorigenic properties in TMZ-R cells and decrease TMZ-R’s ability to generate M2 GAM and glioma stem cells, in association with down-regulation of lncSNHG15 and up-regulation of tumor suppressor miR-627. The level of lncSNHG15/CDK6 may serve as a prognostic marker for TMZ resistance and palbociclib could be used as an adjuvant for overcoming TMZ resistance.

## Additional files

Additional file 1: **Table S1.** Clinical-pathological features of patients in this study. (DOCX 16 kb)

Additional file 2: **Table S2.** Sequence primers used in this study. (DOCX 14 kb)

Additional file 3: **Figure S1.** Full-size blots of Fig. 2e. **Figure S2.** Full-size blots of Fig. 4b. **Figure S3.** Full-size blots of Fig. 6a. (DOCX 1150 kb)

## Data Availability

The datasets used and analyzed in the current study are available from the corresponding author in response to reasonable requests.

## Abbreviations

GBM: Glioblastoma
lncRNAs: long non-coding RNAs
M2-GAMs): M2-polarization of glioma associated microglia
NC: negative controls
TME: tumor microenvironment
TMZ-R: resistant
TMZ-S: temozolomide sensitive
