# Supplementary material for: Modulating lncRNA SNHG15/CDK6/miR-627 circuit by palbociclib, overcomes temozolomide resistance and reduces M2-polarization of glioma associated microglia in glioblastoma multiforme
Source: J Exp Clin Cancer Res. 2019 Aug 28;38:380. doi: 10.1186/s13046-019-1371-0 (PMC6714301; doi:10.1186/s13046-019-1371-0)
Supplement: Supplementary file 1 — Table S1. Clinical-pathological features of patients in this study. (DOCX 16 kb) [file 13046_2019_1371_MOESM1_ESM.docx]

**Additional file 1: Table S1.** Clinical-pathological features of patients in this study

| Clinical-pathological  parameters | N=  40 | SNHG15 expression | | χ2 | *P value* | TMZ responder |
| --- | --- | --- | --- | --- | --- | --- |
|  |  | Low:9 | High:31 |  |  |  |
| Age (years) |  |  |  |  |  |  |
| ≤45 | 17 | 4 | 13 | 1.303 | 0.2537 |  |
| ＞45 | 23 | 10 | 13 |  |  |  |
| Gender |  |  |  |  |  |  |
| Male | 25 | 9 | 16 | 0.223 | 0.6368 |  |
| Female | 15 | 5 | 10 |  |  |  |
| WHO grade |  |  |  | 14.025 | 0.0029 |  |
| I grade | 4 | 2 | 2 |  |  | 2 |
| II grade | 5 | 4 | 1 |  |  | 4 |
| III grade | 14 | 2 | 12 |  |  | 2 |
| IV grade | 17 | 1 | 16 |  |  | 1 |
