# Supplementary material for: Modulating lncRNA SNHG15/CDK6/miR-627 circuit by palbociclib, overcomes temozolomide resistance and reduces M2-polarization of glioma associated microglia in glioblastoma multiforme
Source: J Exp Clin Cancer Res. 2019 Aug 28;38:380. doi: 10.1186/s13046-019-1371-0 (PMC6714301; doi:10.1186/s13046-019-1371-0)
Supplement: Supplementary file 2 — Table S2. Sequence primers used in this study. (DOCX 14 kb) [file 13046_2019_1371_MOESM2_ESM.docx]

**Additional file 2: Table S2. Sequence primers used in this study**

| Gene | Forward | Reverse |
| --- | --- | --- |
| SNHG15 | GCTGAGGTGACGGTCTCAAA | GCCTCCCAGTTTCATGGACA |
| GAPDH | GAAGAGAGAGACCCTCACGCTG | ACTGTGAGGAGGGGAGATTCAGT |
| U6 | CTCGCTTCGGCAGCACA | AACGCTTCACGAATTTGCGT |
| CD163 | TCCACACGTCCAGAACAGTC | CCTTGGAAACAGAGACAGGC |
| CD206 | CAGGTGTGGGCTCAGGTAGT | TGTGGTGAGCTGAAAGGTGA |
| IL-1β | CTGGTGTGTGACGTTCCCATTA | CCGACAGCACGAGGCTTT |
| IL-6 | CCAGTTGCCTTCTTGGGACT | GGTCTGTTGGGAGTGGTATCC |
| IFN-γ | TGT AGC GGA TAA TGG AAC TCT TTT | AAT TTG GCT CTG CAT TAT T |
| TNF-α | CCG AGG CAG TCA GAT CAT CTT | AGC TGC CCC TCA GCT TGA |

|  |  |  |
| --- | --- | --- |
| Si-SNHG15 (1) | UGUAGAAACACUGACGGAUGGCAGG | CCUGCCAUCCGUCAGUGUUUCUAC |
| Si-SNHG15(2) | GAGCAAGUUUGAAACCUCGCUUGUU | AACAAGCGAGGUUUCAAACUUCCUC |
