# Supplementary material for: Modulating lncRNA SNHG15/CDK6/miR-627 circuit by palbociclib, overcomes temozolomide resistance and reduces M2-polarization of glioma associated microglia in glioblastoma multiforme
Source: J Exp Clin Cancer Res. 2019 Aug 28;38:380. doi: 10.1186/s13046-019-1371-0 (PMC6714301; doi:10.1186/s13046-019-1371-0)
Supplement: Supplementary file 3 — Figure S1. Full-size blots of Fig. 2e. Figure S2. Full-size blots of Fig. 4b. Figure S3. Full-size blots of Fig. 6a. (DOCX 1150 kb) [file 13046_2019_1371_MOESM3_ESM.docx]

**SUPPLEMENTARY INFORMATION**

**Modulating lncRNA SNHG15/CDK6/miR-627 circuit by palbociclib, overcomes temozolomide resistance and reduces M2-polarization of glioma associated microglia in glioblastoma multiforme**

Zhenzhe Li^1#^, Jixing Zhang^1#^，Hongshan Zheng^1^，Chenlong Li^1^, Jinsheng Xiong^1^, Weiliang Wang^1^, Hongbo Bao^1^, Hua Jin^1^, Peng Liang^1*^

^1^Department of Neurosurgery, Harbin Medical University Cancer Hospital, Harbin, Heilongjiang 150001, P.R. China

# These authors contributed equally to this work.

* Corresponding Author: Professor. Peng Liang, Department of Neurosurgery, Harbin Medical University Cancer Hospital, No.150 Haping Road, Nangang District, Harbin, Heilongjiang 150001, P.R. China. TEL/Fax:86-451-86298863

E-mail: [liangpengd@yahoo.com](mailto:liangpengd@yahoo.com)


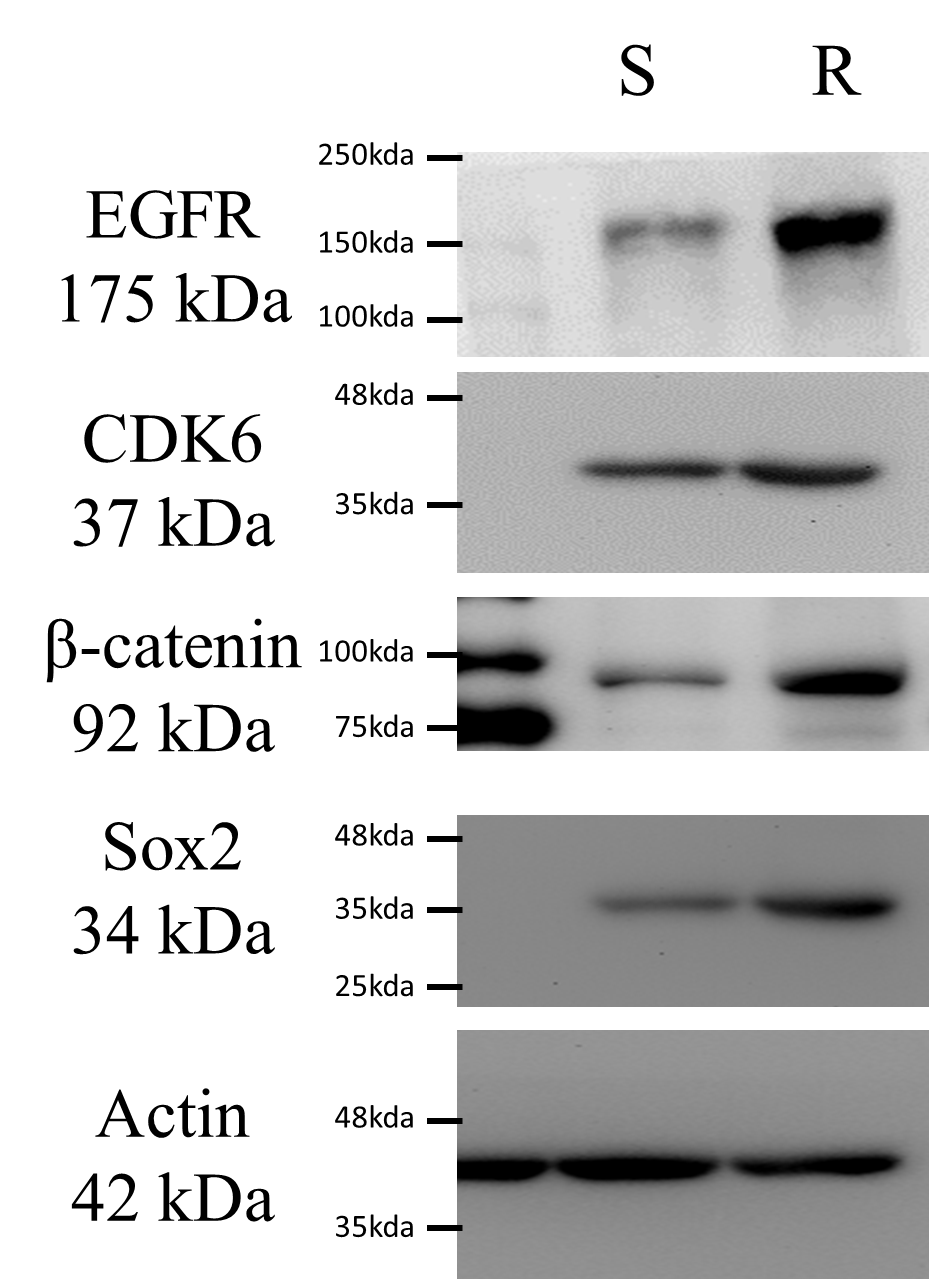


**Supplementary Figure S1.** Full-size blots of Figure 2E


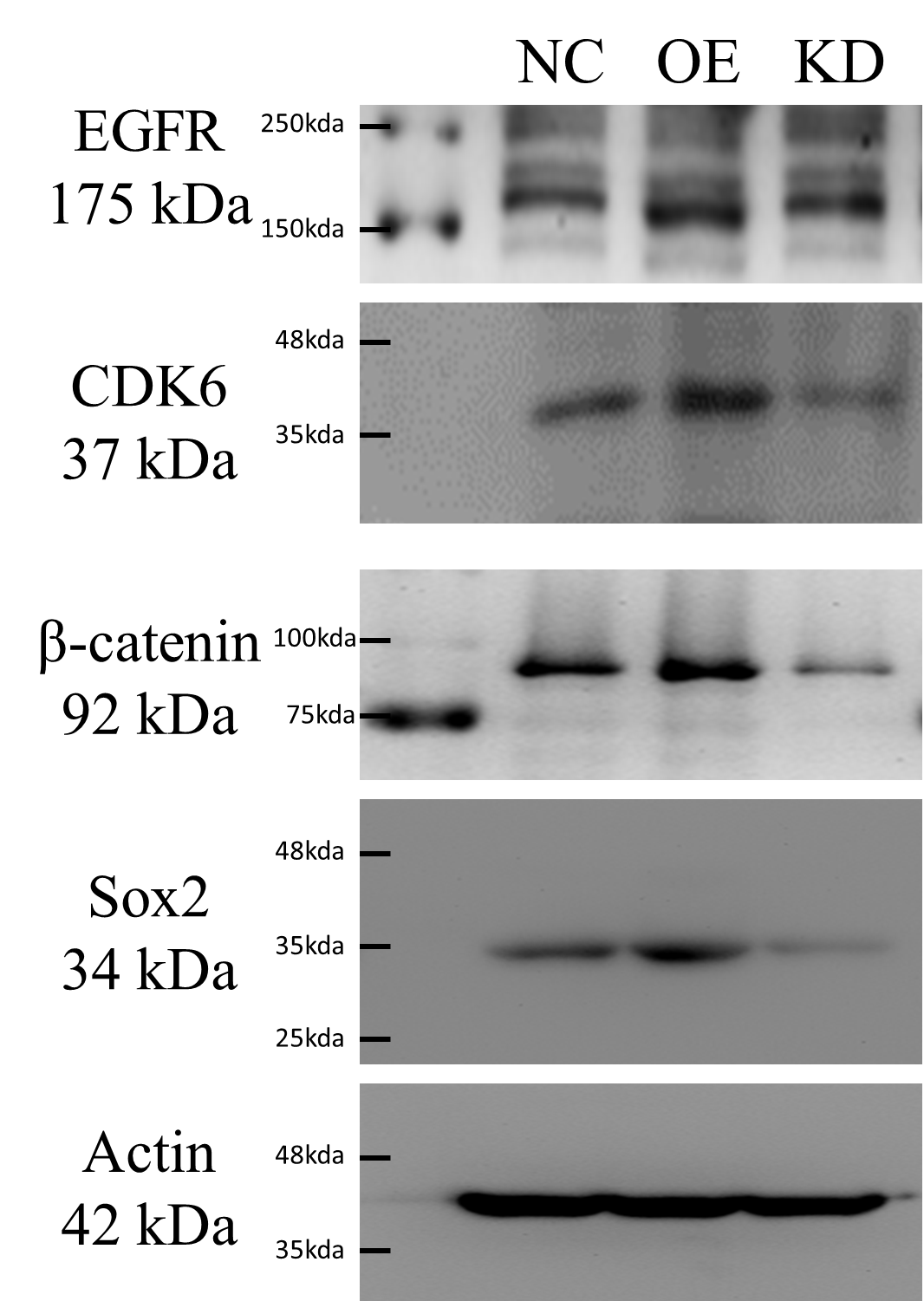


**Supplementary Figure S2.** Full-size blots of Figure 4B


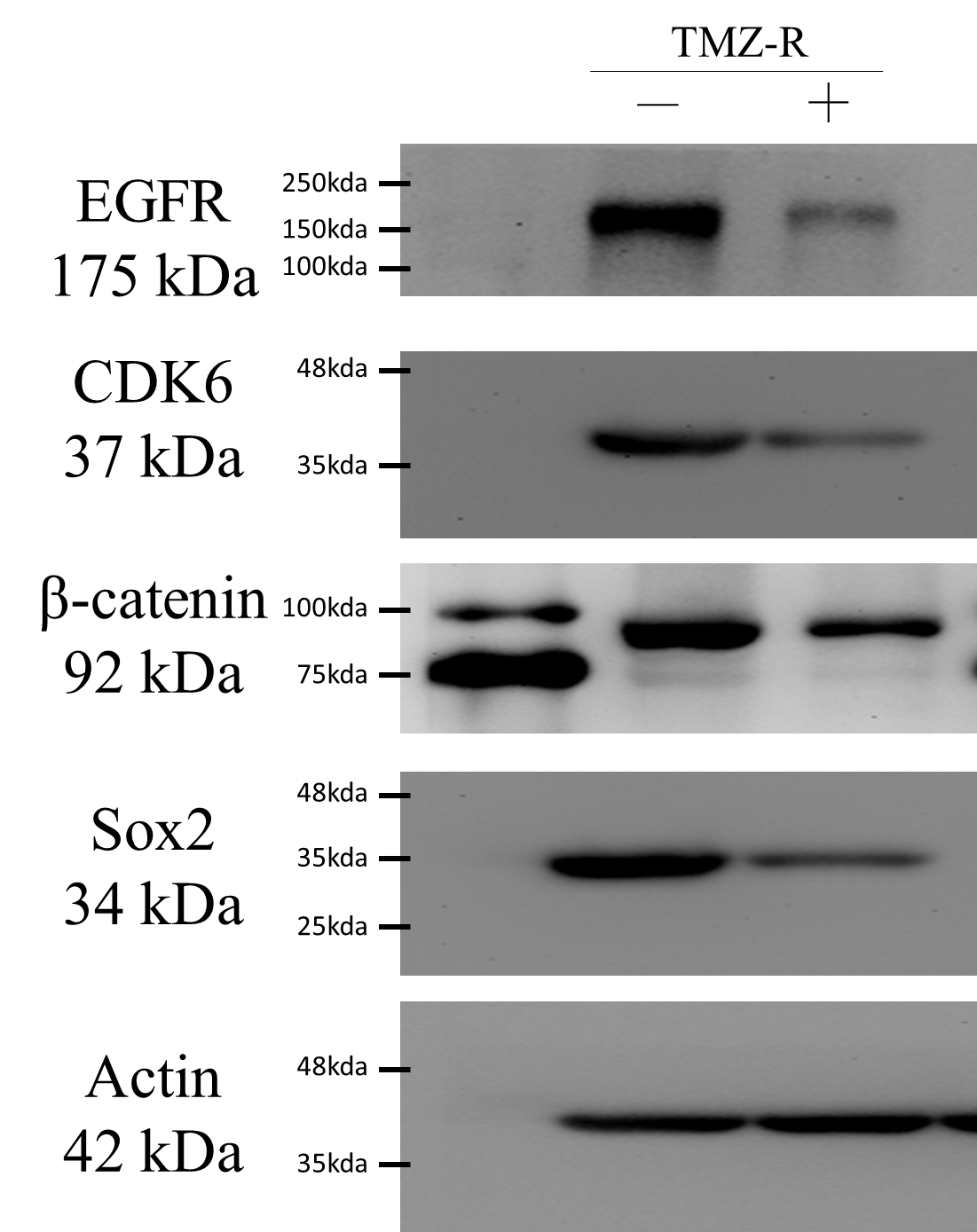


**Supplementary Figure S3.** Full-size blots of Figure 6A.
